# Evaluation of Two Practical Tools to Assess Cognitive Impairment in Aged Dogs

**DOI:** 10.3390/ani12243538

**Published:** 2022-12-15

**Authors:** Susana Le Brech, Marta Amat, Déborah Temple, Xavier Manteca

**Affiliations:** 1 School of Veterinary Medicine, Universitat Autònoma de Barcelona, 08193 Bellaterra, Spain; 2 Animal Welfare Education Centre (AWEC), Edifici Eureka, Campus UAB, 08193 Bellaterra, Spain

**Keywords:** cognitive impairment, aged dogs, assessment tools

## Abstract

**Simple Summary:**

The elderly pet population has increased dramatically in recent decades; consequently, the prevalence of age-related diseases, such as Cognitive Dysfunction Syndrome, has also increased. This syndrome is mainly characterized by cognitive impairment and behavioral changes related to it. The early diagnosis of this condition is important because the sooner treatment is implemented, the better the animal’s response. Diagnostic tools to detect cognitive decline may differ depending on the environment in which the animal lives. In this study, we describe two practical methods for assessing cognitive decline in different environments: (i) a canine cognitive assessment scale for dogs living in a domestic environment and (ii) a practical cognitive test for dogs not living in a domestic environment (e.g., shelters). We also evaluate the effect of age on the results of both tools and, finally, we compare their results. Both methods were found to be practical to perform and their results were related to the age of the animals. However, the results of the scale did not predict the outcome of the test. We suggest that the lack of relationship between test results and scale is probably related to the fact that each tool may be subject to different sources of variability and requires further investigation.

**Abstract:**

Cognitive dysfunction syndrome is the most common cause of cognitive decline in aged dogs. Early diagnosis is crucial because the sooner treatment is implemented, the greater the chance of slowing the progression of the disease. Assessment tools to assess cognitive decline may differ depending on the environment in which the dogs live. The aims of this study were threefold, first, to describe two feasible methods to evaluate cognitive impairment in aged dogs living in different environments: (i) a Canine Cognitive Assessment Scale (CCAS) for dogs living in a home environment and (ii) a practical cognitive test (PCT) potentially useful for dogs not living in a home environment (NHE); second, to assess the effect of age on the outcome of both tools and, finally, to compare the results of the CCAS with those of the PCT. Both methods were found to be practical to perform. Age was found to significantly predict the score obtained by the CCAS (*p* = 0.0011) and the outcome of the PCT (*p* = 0.009). However, the reversal phase from the PCT did not significantly predict the outcomes of the CCAS (*p* = 0.97). Taken together, these findings suggest that the CCAS is a practical method to evaluate age related cognitive changes in owned dogs. The fact that the PCT has not been proven to be related with the CCAS calls into question the use of the PCT as a sensitive tool to assess cognitive impairment. Further studies in this field are suggested.

## 1. Introduction

Cognitive Dysfunction Syndrome (CDS) is an age-related neurodegenerative disease associated with the gradual and progressive loss of cognitive capacity in old dogs [1] and is the most common cause of cognitive decline in aged dogs. CDS can manifest itself through behavioral changes, decreased learning ability and memory, decreased response to stimuli and confusion. The acronym DISHAA is frequently used to describe the main behavioral changes associated with CDS and it refers to disorientation, altered social interactions with people or other pets, altered sleep-wake cycles, house soiling and loss of other learned behaviors, altered activity levels and increasing anxiety [2,3,4,5,6,7,8]. According to several studies, the prevalence of CDS is high, ranging from 14.2% to 68% [3,5,9] and, both the prevalence [9] and severity of symptoms increase with age [3].

Early diagnosis CDS is crucial, because the sooner the treatment is implemented the greater the chance to delay the progression of the disease [10] and improve the quality of life of the affected dogs [11]. From the aforementioned, the need for practical diagnostic tools to allow early detection becomes evident. For a method to be practical, it has to be easy and quick to perform [12]. In the last years, a variety of scales to diagnose, evaluate and determine the prevalence of CDS in dogs have been created [3,4,5,8,13,14]. The information of those tools is based on the dog’s owners’ responses. Owners can provide useful information about their dog’s behavior because most owners have close contact with their dogs. However, many of the scales which are available and have been validated are designed to be completed by specialists, which may limit their usefulness. If the scale can be completed directly by the owner, more cases of cognitive impairment could be detected. On the other hand, some scales are quite long, which makes it less likely that owners will want to complete them. Even when the scale can be practical for owned dogs, they may not be feasible for other populations of dogs, especially for those not living in a home environment (NHE) such as shelter dogs, working dogs and laboratory dogs, among others. In these groups of dogs, the caretakers may not be able to respond to the questions relating to these scales because they are usually based on contextualized situations in the domestic environment that may not be applicable to NHE dogs. For this reason, another practical tool to assess cognitive impairment for NHE dogs is also needed.

Cognitive tests represent a method of direct evaluation of dogs that allows for the assessment of their cognitive state. Several cognitive tests that measure the learning abilities and spatial memory of dogs have been used and provide valuable information on cognitive impairment [15,16,17,18,19]. It was observed, for example, that aged dogs usually do not demonstrate decline in simple tasks as a spatial memory task, such as when they are repetitively rewarded for approaching one of two different locations [20]. However, if the reward contingencies are reversed once the dog has learned the simple discrimination task so that the dog must learn to respond selectively to the location that was not previously rewarded, aged dogs require significantly more attempts to learn to respond consistently to the new rewarded object than young dogs [15,21]. One of the main advantages of the tests is that they represent a direct method of assessment; however, they have the disadvantage that most of them must be performed in laboratory conditions and require a lot of time to train the dogs to do them [15], which makes them difficult to be applied in a field situation. Some tests have been adapted to assess aged, owned dogs in the clinical setting [22,23]; however, they still required at least one hour to be performed, so they may not be practical in all cases. For this reason, a more practical test would be needed to evaluate cognitive impairment in NHE dogs.

Finally, it must be considered that many medical conditions can affect behavior. This is particularly relevant in aged dogs where diseases and sensory impairments are more prevalent. For this reason, it is important to consider the medical history of the dogs when interpreting the results of the behavioral assessment tools as, for example, in the case of the scales, many of the questions can be affected by both medical, sensory or cognitive problems.

The aims of this study were, first to describe two practical methods to evaluate cognitive impairment in aged dogs living in different environments. A Canine Cognitive Assessment Scale (CCAS) was described to evaluate dogs living in a home environment. A Practical Cognitive Test (PCT) that would be potentially useful for assessing the cognitive status in dogs not living in a home environment was also evaluated. Furthermore, we assessed the effect of age on the outcome of both tools. Finally, we compared the results of the CCAS with those of the PCT. We hypothesized that the age would be related to both assessment tools and that their outcomes would also be related.

## 2. Materials and Methods

### 2.1. Study 1: Canine Cognitive Assessment Scale (CCAS)

#### 2.1.1. Subjects

This study was carried out between June and November 2018. The dogs were regular patients of the Veterinary Hospital of the Universitat Autònoma de Barcelona that were not referred for behavioral consultations. The dogs were at least 8 years old. A total of 100 questionnaires were obtained. The mean age (mean ± SD) of the dogs was 10.5 ± 2.12 (range: 8–18 years). The mean weight was 20.2 ± 16.8 (range: 2–49.5 kg). There were 74 pure bred dogs and 26 crossbred dogs (Table 1). Male dogs (52%) slightly outnumbered female dogs (48%). Most dogs were neutered: 85.4% of females and 61.5% of males.

#### 2.1.2. Description of the Canine Cognitive Assessment Scale (CCAS)

The CCAS (Table 2) was adapted and modified from questionnaires proposed by different studies [4,8,14], based on the signs already reported in literature [1] and on the clinical expertise of the authors of this work. The CCAS includes 17 items grouped into 6 different sections related to the type of changes in the dogs’ behavior: Disorientation, Sleep–Wake Cycle, Social Interaction, Learning and Memory, Activity Level and Anxiety. The frequency of the behaviors was assessed using a 4-point scale: Never (0 points)–Once a month (1 point)–Once a week (2 points)–Almost every day (3 points). Based on the score obtained and experts’ interpretation of these outcomes, dogs were classified in one of three categories of cognitive state: Normal Ageing (NA) 0–7 points, Mild/Moderate Cognitive Impairment (MCI) 8–40 points and Severe Cognitive Impairment (SCI) 40–69 points. The owners had to indicate only what they had observed in the last 6 months.

The scale was filled out by the owners in the waiting room. A requirement to be included in the study was that the dogs had to live in close contact with their owners, so that the owners could adequately assess behavioral changes in their dogs. The researcher asked owners about general dog information (age, sex, reproductive status, breed, weight, body condition, current diet and current medications) and about medical history, sensory deficits and pharmacological treatment. The dogs’ hospital medical records were also available to the researchers. Dogs with chronic diseases or under drug treatment were not excluded if their actual condition was good and it was not expected to interfere with their cognitive status. None of the dogs included were patients of the ethology service.

### 2.2. Study 2: Practical Cognitive Test (PCT)

#### 2.2.1. Subjects

This study was also carried out at the Faculty of Veterinary Medicine of the Autonomous University of Barcelona from September to November 2019. The 40 dogs included were not the same as in the Study 1. They were recruited from veterinary students, teaching staff and friends.

At the beginning of the study, the dog owners had to complete the CCAS. Moreover, as in the Study 1, we obtained general information (age, sex, reproductive status, breed, weight, body condition, current diet and current medications) and about medical history, sensory deficits and pharmacological treatment. Dogs with chronic diseases or under drug treatment were not excluded if their actual condition was good and it was not expected to interfere with their cognitive status. Fearful or aggressive dogs were not included in the study.

The dogs were at least 8 years old and belonged to different breeds (Table 3) or were mixed breed. The mean age (mean ± SD) of the dogs was 11.6 ± 1.9 (range: 9–16 years). The mean weight was 24.5 ± 17.3 (range: 3.5–38 kg). Twenty-five of the dogs were females, 18 of which were spayed and 15 were males, 8 of which were neutered.

#### 2.2.2. Description of the PCT

**Figure 1:**
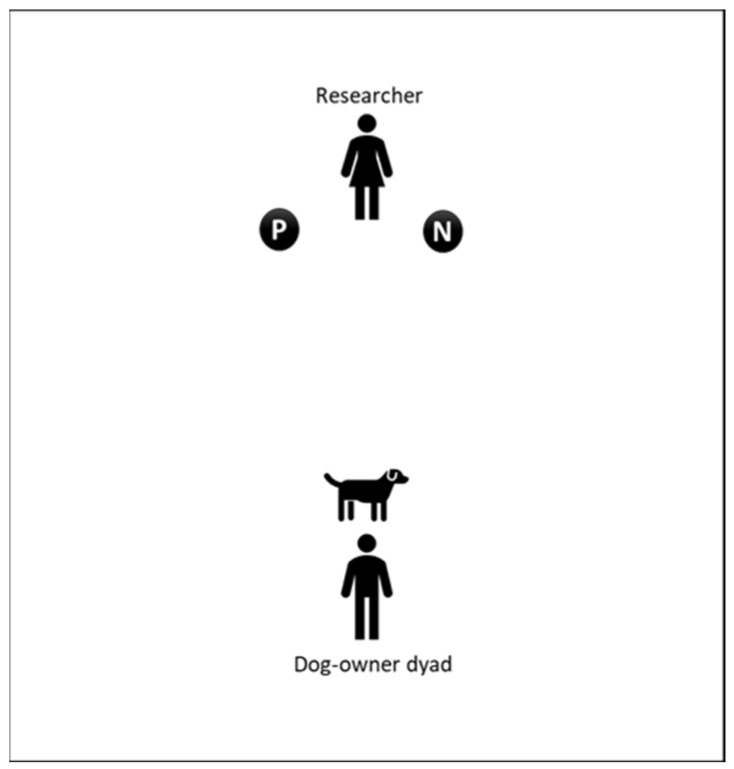
Room set up: The owner and the dogs were at one side of the room and the researcher was in a crouched position opposite to them. The circles in front of the experimenter mark the positions of the experimenter hands where they had a treat (P) or did not have a treat (N).

After completing the CCAS, the forty dogs were assessed through the PCT. The test was adapted and simplified from (Piotti et al.) [23]. It was carried out in a closed room (Figure 1) and consisted of two tasks: Discrimination Learning and Reversal Learning.

Discrimination Learning. Dogs had to associate the presence (Positive) or absence (Negative) of food with the specific location (right or left). The food was placed in one hand of the experimenter, either the left or right hand. For 20 dogs, the positive location was on the left and the negative was on the right, while the opposite was true for the other half. To control for odor cues, both hands of the experimenter were equally smeared with food reward (sausage or ham) prior to testing. The experimenter stood approximately 1 m from the dog-owner dyad with hands closed and behind the body. To avoid pointing at the dog, the researcher avoided looking directly at the dog during the test.

The owner held the dog to keep it in front of the experimenter (on a spot marked on the floor) and did not release the dog until the experimenter requested them to do it.

At the beginning of each trial, the experimenter presented both hands closed and asked the owner to release the dog. The dog had to choose a hand, if he chose the positive hand, the experimenter opened it and gave the treat to the dog (the dog scored a point). If the dog approached the negative hand, the experimenter would not open it and would place both hands behind the body until the next trial (the dog scored no points). Each session consisted of ten trials. The dog was considered to have learned to associate the food with the positive position when it met the criteria of 9 out of 10 correct choices (9 points out of 10) in a single session or 8 out of 10 correct choices (8 points out of 10) in two sessions, consecutively. The maximum number of sessions was 5.

Reversal learning. This task was identical to the discrimination learning task, except that the P and N were reversed. For example, if the positive stimulus was on the left, now it will be on the right.

The duration of the complete test was 10 min, approximately.

### 2.3. Statistical Analysis

SAS package (version 9.4, SAS Inst. Inc., Cary, NC, USA) was used for all statistical analysis. The total behavioral obtained from each of the 100 dogs evaluated with the CCAS was calculated by summing the scores obtained in each question. Questions belonging to the section Disorientation were summed twice. A Wilcoxon signed rank test was used to investigate whether there were significant differences between the scores obtained by each category of the CCAS.

Multiple regression models by means of the GLM procedure were performed to develop prediction equations on a subsample of 40 dogs, which performed both CCAS and PCT. A first model was used to analyze the influence of the age of the dog, the gender and reproductive status on the score obtained by the CCAS. A second model was used to determine the effect of the age of the dog, the gender and reproductive status on the outcomes of the PCT (number of sessions and number of errors at the reversal test). Finally, a third multiple regression model was used to test whether the outcomes of the reversal phase from the cognitive test significantly predicted the score obtained by the CCAS scale. A *p*-value of 0.05 was considered significant for all analyses.

### 2.4. Ethics Statement

The procedures applied in both studies complied with the regulations of the European Data Protection Regulation (RGPD) and the Organic Law on Protection of Personal Data and Guarantee of Digital Rights (LOPDGDD) and followed the ethical guidelines of the Autonomous University of Barcelona and the Animal Protection Law of Spain. In the Consent Form, participants were informed about the identity of the researchers, the general aim of the study, procedure, location, expected time commitment of the experiment, the handling of personal and research data and data reuse. The information included the participant’s right to withdraw their consent at any time. Participants could at any point decline to participate and could request for their data not to be used and/or deleted after they were collected during the experiments.

## 3. Results

### 3.1. Study 1: Canine Cognitive Assessment Scale (CCAS)

Of the total of 100 dogs that participated in the study, 75 dogs obtained a score that corresponded to NA and 25 dogs obtained a score corresponding to MCI (Mild Cognitive Impairment). None of the dogs obtained a score corresponding to SCI (Severe Cognitive Impairment).

**Figure 2:**
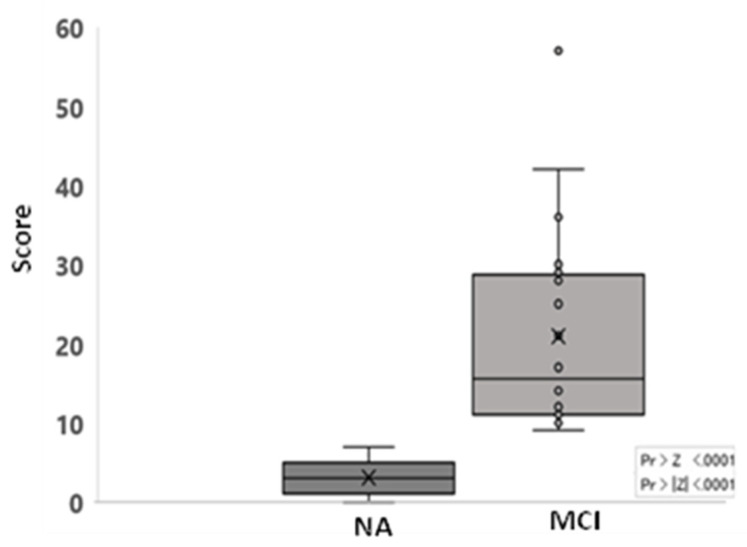
Score of the CCAS according to the cognitive impairment. Normal ageing (NA, n = 75); Mild/Moderate Cognitive Impairment (MCI, n = 25).

The scores obtained by dogs classified as having MCI were significantly different from the score obtained by dogs in the group of NA (*p* < 0.0001) (Figure 2).

The mean score of the CCAS was 12 ± 13.1 (median: 9; Min/Max [0/57]). Multiple regression was used to analyze the influence of the age of the dog, the gender and reproductive status on the score obtained by the CCAS. It was found that the age significantly predicted the score obtained by the scale (R^2^ = 28%, β = 3.5, *p* = 0.0011). The gender (*p* = 0.53) and reproductive status (*p* = 0.51) did not significantly predict the score obtained by the scale.

### 3.2. Study 2: Practical Cognitive Test (PCT)

Of the 40 dogs that participated in the study, one dog was excluded from the analysis of the test results because he displayed a high level of fear and could not complete the test. Twenty dogs obtained a CCAS score corresponding to NA and 19 dogs to MCI. The mean, standard deviation, median, minimum and maximum of the total number of sessions and errors during both tasks are summarized in Table 4.

**Figure 3:**
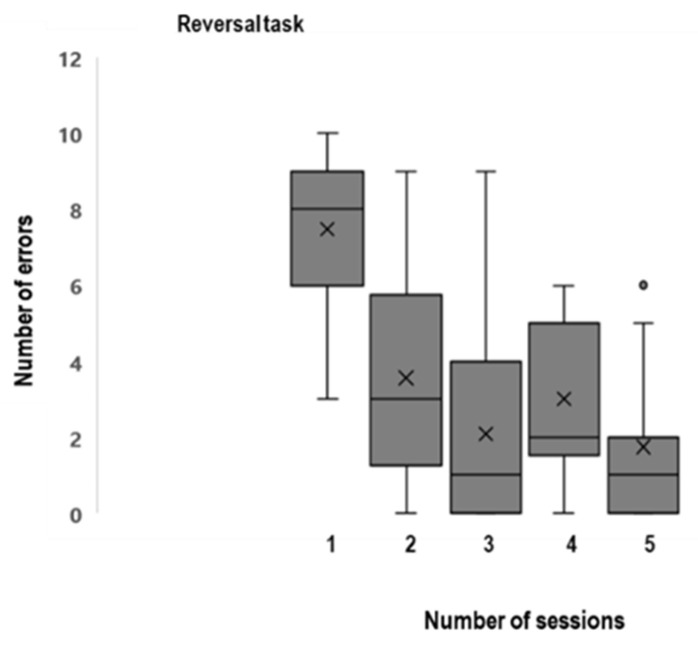
Number of sessions and errors of the Reversal task (n = 39).

A multiple regression was used to determine the effect of the age of the dog, the gender and reproductive status on the outcomes of the cognitive test (number of sessions and number of errors at the reversal task) (Figure 3). It was found that the age significantly predicted outcomes of the cognitive test (number of sessions: R^2^ = 20%, β = 0.24, *p* = 0.009; number of errors: R^2^ = 23%, β = 1.91, *p* = 0.005). The gender (*p* = 0.36) and reproductive status (*p* = 0.27) did not significantly predict the outcomes of the cognitive test.

### 3.3. Relationship between CCAS and PCT

Multiple regression was used to test whether the outcomes of the reversal phase from the PCT significantly predicted the score obtained by the CCAS. It was found that the reversal phase from the PCT (number of sessions: β = 0.24, *p* = 0.50; number of errors: β = 0.02, *p* = 0.97) did not significantly predict the outcomes of the CCAS.

## 4. Discussion

With the advances in veterinary medicine and nutrition, the elderly pet population has increased dramatically in recent decades. Consequently, the prevalence of diseases related to old age, such as CDS, have also increased. In the first part of our study, we observed that 25% of the old dogs presenting to the Veterinary Hospital presented a score compatible with CDS. Although the sample was quite small, this result is in accordance with other studies that also found a high prevalence of this syndrome [4,5,9]. Similarly, to what was observed in [4,9], none of the owners of dogs exhibiting signs of CDS had reported these signs to the veterinarian. Some of the reasons that may contribute to the low reporting rate would include a lack of awareness of this syndrome and the misconception that behavioral changes indicative of CDS are part of “normal ageing” [20]. Although CDS is a degenerative disease that has no cure, the implementation of management strategies can slow the progression of the symptoms and significantly improve the quality of life of the patients [24]. The sooner the treatment is implemented, the better the response, hence the importance of early detection of the symptoms. Treatment options for CDS includes dietary and pharmacological intervention. Nutritional supplementation blends, for instance, have been demonstrated to provide cognition-improving effects and can be used to delay cognitive ageing [25,26]. Drugs such as selegiline have demonstrated an improvement of the clinical signs of CDS and an improvement in working memory [2,27,28]. Additionally, changes in the environment may be extremely helpful, especially when combined with dietary management [29].

One of the aims of this study was to describe two feasible methods to evaluate cognitive impairment in aged dogs living in different environments. These tools would allow for the early detection of dogs with some degree of cognitive impairment. A Canine Cognitive Assessment Scale (CCAS) was described to evaluate dogs living in a home environment. The CCAS contain only 17 questions designed to be responded by owners, which makes it quick and easy to be completed by them and can be used on a day-to-day basis in veterinary clinics to proactively detect signs of cognitive impairment in owned dogs. The scores obtained by dogs classified as having Mild/Moderate Cognitive Impairment were significantly different from the score obtained by dogs in the Normal Ageing group. None of the 100 dogs that participated in the first experiment and only two that participated in the second one demonstrated a score compatible with Severe Cognitive Impairment. One possible explanation for this last result could be that dogs with Severe Cognitive Impairment may be in too poor health with cognitive impairment per se and/or other medical problems, that they did not meet the inclusion criteria to participate in the study. Additional studies are required to confirm this hypothesis.

The CCAS, however, may not be feasible to other populations of dogs such as NHE dogs, such as shelter dogs. For this population of dogs, we described PCT to evaluate the cognitive status of dogs. This test can also be applied to dogs living in a home environment, but scales will likely be more practical for these dogs. Most cognitive tests published in the literature are performed in laboratory conditions and required a previous training of the dogs. In this study, we adapted the test proposed by [23] that can be performed in a short time frame, and we obtained an even shorter and more practical test that could be performed in any population of dogs. The test we used consisted of two tasks: Discrimination Learning and Reversal Learning. All dogs, except one that displayed a high level of fear, completed the test. The fact that 97.5% of the owned dogs that participated in this study successfully completed the test may indicate that this short test could be also a promising and feasible tool to evaluate the cognitive states in NHE dogs. Another advantage of the use of cognitive tests is that the dogs are highly motivated to perform them because they are rewarded with food and they have extra contact to people, which would represent a kind of environmental enrichment for NHE dogs, such as shelter dogs.

The effect of age on the outcome of both assessment tools was investigated, and it was found that the age significantly predicted the score obtained by the scale and the outcome of the cognitive test. Age-related impairment in cognitive function was found in pet [3,5,6,23,30] and laboratory dogs [15,31].

At the beginning of the study, we hypothesized that the outcome of the test would predict the scale score, but no such relationship was found. Dogs with true MCI will have impaired executive functions, which should be confirmed by the reversal task of PCT. However, our results demonstrated that the reversal task did not predict the CCAS score. This could suggest that the CCAS scores failed to reflect the cognitive status of the dogs, or that the reversal task setup did not measure executive function in the dogs. In fact, it is possible that each tool measures different parameters that could be associated with different sources of variability. Like other scales, the CCAS score is based on behavioral changes that occur in a dog’s daily life and that may be related to cognitive decline or that may be the result of other age-related changes (e.g., medical conditions and sensory disabilities) [1]. In this sense, we suggest that the CCAS scale provides information that is more relevant to assess the quality of life of senior dogs than that provided by tests.

One of the advantages of this study was that we have the medical records of the dogs that participated in this study, and we have contact with the dogs; however, it is still possible that some subtle symptoms may have gone unnoticed and may have affected the results of the scale. Another source of variability associated with the scale arises from the fact that they are completed by the owners, and, although the questions are focused on specific parameters, they may still have differences in perception between them.

As for the test, it represents a direct method of assessing cognition, but can also be subject to variability, especially when performed with dogs from different sources (e.g., non-laboratory dogs), as in this case. For example, dogs that are highly motivated by treats may be more enthusiastic about the test, and this could have influenced the results. Fear, distress and a negative cognitive bias could also be sources of variation [32]. In fact, it has been proposed that learning ability be used to assess the well-being of dogs [33]. Although only one dog was unable to complete the test because it was too afraid to do so, this does not rule out subtle differences in the level of fear, stress, or cognitive bias in the rest of the dogs. Previous experiences in performing learning tasks could also have influenced the results. In another study, pre-training was found to improve dogs’ problem-solving ability [34]. The dogs’ previous experiences with training were not taken into account as an inclusion criterion in this study. Other factors such as breed, size and weight can be a source of variability [35] both in the test and in the scale. Finally, it should be considered that, in other studies, a decrease in spatial function was observed before other cognitive deficits emerged [19,23,36], and as such the PCT may well detect subtle cognitive changes that may be difficult to identify in the CCAS used in dogs in the home environment [1]. It is possible that in our study the PCT detected changes that were not detected by the scale. This does not mean that the scale is not sensitive for assessing cognitive decline, but rather that the test would likely detect more subtle changes that the scale does not detect. More studies are needed to test this hypothesis.

The lack of an already validated golden standard against which both tools can be compared is a limitation of this study. Further research would be needed to clear up this limitation. Finally, it would be interesting to include dogs from other age categories, rather than focusing on old dogs, to analyze the effect of age in more detail.

## 5. Conclusions

CDS is the most common cause of cognitive decline and a highly prevalent disease in older dogs. Early diagnosis is essential to relieve symptoms, increase the quality of life of affected patients and slow down the progression of the disease. Practical evaluation methods are fundamental tools to increase the diagnosis rate. Two different tools to evaluate cognitive impairment in aged dogs were described. Age was found to predict the results of both tools. The CCAS was found to be a practical method for assessing cognitive impairment in owned dogs. Despite the relationship of the PCT with aging, it has not proven to be related with the CCAS. This calls into question the use of the PCT as a sensitive tool to assess cognitive impairment.

## Figures and Tables

**Table 1:** Breeds of dogs that participated in the Study 1.

Breed	No.
American Stafforshire Terrier	1
Andalusian Hound	1
Beagle	2
Belgian Sheepdog	5
Maltese	6
Boxer	4
Brittany dog	4
Poodle	1
Pug	1
Chihuahua	1
Cross breed	26
French Bulldog	2
German Shepherd	7
Golden Retriever	7
Great Pyrenees	1
Greyhound	1
Havanese Dog	1
Siberian Husky	3
Labrador Retriever	5
Pomeranian	2
Pyrenean Mountain Dog	1
Schnauzer	2
Shar-Pei	1
Shih Tzu	1
Sighthound	1
Spanish Water Dog	2
Spitz	2
Staffordshire Bull Terrier	2
West Highland White Terrier	1
Whippet	2
Yorkshire Terrier	4

**Table 2:** Canine Cognitive Assessment Scale (CCAS). Frequency of the behavior observed: Never (0 point)–Once a month (1 point)–Once a week (2 points)–Almost every day (3 points). The scores of Section 1 sum twice. Clinical stage: Normal Ageing (score 0–7), Mild cognitive impairment (score 8–40) and Severe cognitive impairment (score 41–69).

Section 1. Disorientation (score × 2)
Stares intently where there is nothing visible.
2. Does not remember its way back home.
3. Becomes stuck behind objects or furniture.
4. Stays on the wrong side of the door.
5. Does not respond to certain stimuli to which it used to respond (for example, doorbell).
6. Does not give any signal when it wants to go out.
Section 2. Social Interaction
7. Does not recognize familiar people.
8. Does not recognize familiar animals.
9. Shows more signs of fear or aggression towards people and/or other dogs than it used to be.
Section 3. Sleep–Wake Cycle
10. Walks during the night (without an obvious reason) when it did not use to do this.
11. Vocalizes (barks, whines) during the night (without an obvious reason), when it did not use to do this.
Section 4. Learning and Memory
12. Urinates and/or defecates in new (inappropriate) places (when it did not use to do it).
13. Finds it difficult to respond to previously learned commands.
Section 5. Activity Level
14. Is less active or playful than it used to be.
15. Shows repetitive behaviors (chases own tail, snaps at “invisible” flies, etc.).
16. Walks without obvious purpose.
Section 6. Anxiety
17. Shows more signs of anxiety when separated from its owners than before (main signs of anxiety are shaking, shivering, or trembling, excessive salivation, restlessness/agitation/pacing, whining and loss of appetite).

**Table 3:** Breeds of dogs that participated in the Study 2.

Breed	No.
American Stafforshire Terrier	1
Andalusian wine-cellar rat-hunting dog	2
Beagle	1
Boxer	1
Coton de Tuléar	1
Cross bred	11
English Shepherd	1
Fawn Brittany Griffon	1
German Spitz	1
Golden Retriever	4
Labrador Retriever	1
Lhasa apso	1
Maltese	5
Miniature Schnauzer	2
Pug	1
Scottish Terrier	1
Shih Tzu	2
Valencian rat hunting dog	1
Yorkshire Terrier	2

**Table 4:** Description of the mean, standard deviation, median, minimum and maximum of the Discriminant and Reversal tasks.

	Total Number of Sessions					Total Number of Errors				
	Mean	SD	Median	Min	Max	Mean	SD	Median	Min	Max
Discriminant task	2.0	1.08	2	1	5	4.9	5.77	3	0	25
Reversal task	3.4	1.18	3	2	5	14.1	8.10	12	14	38

## Data Availability

Not applicable.
